# The Left-Side Bias Is Reduced to Other-Race Faces in Caucasian Individuals

**DOI:** 10.3389/fpsyg.2022.855413

**Published:** 2022-04-25

**Authors:** Jing Kang, Chenglin Li, Werner Sommer, Xiaohua Cao

**Affiliations:** ^1^Department of Psychology, Zhejiang Normal University, Jinhua, China; ^2^Department of Biological Psychology and Cognitive Neuroscience, Friedrich Schiller University Jena, Jena, Germany; ^3^Institut für Psychologie, Humboldt-Universität zu Berlin, Berlin, Germany; ^4^Key Laboratory of Intelligent Education Technology and Application of Zhejiang Province, Zhejiang Normal University, Jinhua, China

**Keywords:** facial familiarity, other-race face processing, face inversion, cognition, left-side bias

## Abstract

One stable marker of face perception appears to be left-side bias, the tendency to rely more on information conveyed by the left side of the face than the right. Previous studies have shown that left-side bias is influenced by familiarity and prior experience with face stimuli. Since other-race facial recognition is characterized by reduced familiarity, in contrast to own-race facial recognition, the phenomenon of left-side bias is expected to be weaker for other-race faces. Among Chinese participants, face inversion has been found to eliminate the left-side bias associated with own-race faces. Therefore, it is of interest to know whether face inversion influences left-side bias for non-Chinese research participants and can be generalized across own- and other-race faces. This study assessed 65 Caucasian participants using upright and inverted chimeric Caucasian and Asian faces in an identity similarity-judgment task. Although a significant left-side bias was observed for upright own-race faces, this bias was eliminated by facial inversion, indicating that such a bias depends on the applicability of configural processing strategies. For other-race faces, there was no left-side bias in the upright condition. Interestingly, the inverted presentation yielded a right-side bias. These results show that while left-side bias is affected by familiarity differences between own- and other-race faces, it is a universal phenomenon for upright faces. Inverted presentation strongly reduces left-side bias and may even cause it to revert to right-side bias, suggesting that left-side bias depends on configural face processing.

## Introduction

Faces are both common and special objects in our daily lives. Most humans can be considered face experts, as they can quickly spot a specific face in a crowd and discriminate between hundreds of faces, even at a distance, in poor lighting, or after a long time (Bahrick et al., 1975; Mondloch et al., 2010). Interestingly, however, the two halves of the face are not of equal relevance. People generally rely more on information conveyed by the left side of the face (from the viewer's perspective). When judging faces or carrying out visual searches, the left side is generally inspected first (Guo et al., 2009) and for a longer period of time than the right side (Ricciardelli et al., 2002; Butler et al., 2005; Butler and Harvey, 2006; Guo et al., 2012). These phenomena, which have been termed “left-side bias” (Wolff, 1933), are considered stable markers of perceptual expertise in face processing (Hsiao and Cottrell, 2009). The left-side functional bias for faces is considered to be due to a right hemisphere specialization for face processing, especially in the right fusiform face area, as demonstrated in behavioral studies (e.g., Bourne, 2008) and neuroimaging studies (e.g., Sergent et al., 1992; Thome et al., 2022). Behavioral evidence has revealed a left-side bias in facial-identity recognition (Gilbert and Bakan, 1973), gender decisions (Butler and Harvey, 2005, 2008), emotion judgments (Bourne, 2008, 2011), age judgments (Burt and Perrett, 1997), attractiveness evaluation (Heath et al., 2005), and social touch (i.e., left-cradling bias; Malatesta et al., 2020), as have electrophysiological (Yovel et al., 2003) and neuroimaging (Yovel et al., 2008; Harrison and Strother, 2021) investigations. Initially, researchers investigated left-side biases for face halves, using chimeric faces to establish asymmetry of perception (Levy et al., 1983). Recently, studies on left-side face bias, most of which have used chimeric faces composed of left or right halves of the face, combined with their mirror images, have shown that left-side chimeras are judged to be more similar to the original face than right-side chimeras in facial-identity tests (Gilbert and Bakan, 1973; Rhodes et al., 1990; Brady et al., 2004, 2005; Coolican et al., 2008; Li et al., 2021).

Most previous studies on left-side bias have used own-race faces (e.g., Gilbert and Bakan, 1973; Butler and Harvey, 2005, 2008; Coolican et al., 2008). For example, Gilbert and Bakan (1973) used a facial-identity judgment task involving Caucasian faces to test Caucasian adults, finding a strong left-side bias. Similar results have been obtained in studies involving Caucasian children (Aljuhanay et al., 2010; Balas and Moulson, 2011; Proietti et al., 2015), young adults (Burt and Perrett, 1997; Butler and Harvey, 2005; Butler et al., 2005), and older adults (Levine and Levy, 1986; Coolican et al., 2008). Asian participants have also shown a left-side bias in relation to own-race adult faces (Chung et al., 2017; Li and Cao, 2017; Li et al., 2018).

In the field of face perception, the participants' degree of familiarity with a given category of faces has been shown to be important. First, among various familiar faces, one's own face (self-face) and those of one's family members and friends are recognized more quickly and accurately than unfamiliar adult faces (Hancock et al., 2000; Herzmann et al., 2004), presumably because they are overlearned. Second, in relation to own-age face stimuli, people have more experience with the faces of people their own age rather than younger or older faces; this is widely thought to explain the own-age effect, the advantage that people experience in recognizing/memorizing own-age faces as opposed to other-age faces (Kuefner et al., 2008; Harrison and Hole, 2009; Rhodes and Anastasi, 2012). Third, own-race faces are generally more familiar than other-race faces, a common theoretical finding in research on the own-race face advantage (Malpass and Kravitz, 1969; Brigham and Malpass, 1985; Meissner and Brigham, 2001; Rhodes et al., 2006). Interestingly, higher levels of face familiarity induce a stronger left-side bias (Balas and Moulson, 2011; Proietti et al., 2015). For instance, Brady et al. (2005) investigated the effect of familiarity on left-side bias in a facial-identity judgment task that involved the participants' own faces (self-faces) and those of friends and strangers. They found a stronger left-side bias for self-faces and faces of friends. Similarly, Proietti et al. (2015) found a stronger left-side bias for own-age adult faces as opposed to other-age infant faces.

The findings above, which involve familiar vs. unfamiliar adult faces and own- vs. other-age faces, indicate that face familiarity and exposure or experience play an important role in left-side bias. However, it is not yet clear whether other-race faces reduce left-side bias relative to own-race faces. Compared to other-race faces, own-race faces are much more frequently seen in daily life. Although previous studies have demonstrated a right hemisphere advantage for own-race faces (Turk et al., 2005; Hellige et al., 2010; Davis et al., 2016; Malatesta et al., 2021; but see Hugenberg et al., 2010; Prete and Tommasi, 2019), this likely reflects a greater involvement of the right hemisphere in configural face processing, which tends to elicit a more significant left-side bias. To the best of our knowledge, only two studies have tested left-side bias using both own- and other-race faces. Rhodes et al. (1990) have reported a significant left-side bias among Chinese participants for both Chinese (own-race) and Caucasian (other-race) faces, while Caucasian participants showed a left-side bias for Caucasian (own-race) faces only. Recently, Li et al. (2021) tested Chinese participants, using Chinese and Caucasian faces in both upright and inverted conditions, identifying a left-side bias for both own- and other-race upright faces. However, they found no left-side bias when the same faces were inverted. Thus, Rhodes et al. (1990) and Li et al. (2021) have shown that both Chinese and Caucasian participants exhibit clear left-side biases when processing upright own-race faces and that Chinese participants exhibit left-side bias when processing upright other-race faces.

Interestingly, Li et al. (2021) found no left-side bias when Chinese participants processed inverted own- or other-race faces. In addition, previous studies have shown that the behavior and underlying neural mechanisms associated with inverted face processing differ from those associated with upright face processing (Sergent, 1984; McCarthy, 1999; Itier et al., 2006; McKone et al., 2013). Face inversion alters global facial configuration, although it does not change image symmetry along the vertical axis or the local image properties of individual facial features (i.e., local contrast between eyes and mouth). For this reason, inverted faces not only serve as ideal control images for upright faces (Crookes et al., 2013) but can also help identify the mechanisms underlying left-side bias. However, no studies have investigated left-side bias among Caucasian participants processing other-race inverted faces thus far. It remains unknown whether the absence of left-side bias in inverted faces, as observed in Chinese participants, can be generalized to other ethnicities.

This study has investigated left-side bias in relation to own- and other-race faces with upright and inverted orientations in Caucasian participants. Based on the studies discussed above, which indicate a stronger left-side bias for more familiar faces (Brady et al., 2005; Balas and Moulson, 2011; Proietti et al., 2015), and the lack of any significant left-side bias when Chinese participants process inverted faces (Li et al., 2021), we have hypothesized that (1) Caucasian participants will have a stronger left-side bias effect for own-race faces than for other-race faces in the upright condition and (2) the left-side bias effect will be eliminated or reduced for inverted faces.

## Methods

### Participants

A total of 65 healthy German participants (43 women; *M* = 23.65 years of age, *SD* = ± 3.84, range 18–35 years) were recruited from the nearby Humboldt University of Berlin. They were divided into two groups, with 34 participants (24 women) allocated to the upright condition and 31 participants (19 women) allocated to the inverted condition. A handedness questionnaire (Oldfield, 1971) was used to ascertain that 62 participants were right-handed, one was left-handed, and two were ambidextrous. All participants reported normal or corrected-to-normal vision. Ethical approval was obtained from the Zhejiang Normal University Ethics Committee, and all participants provided their written informed consent.

### Stimuli

The stimuli included 40 images of adult Chinese faces (20 female faces) (Li and Cao, 2017) and 40 images of adult Caucasian faces (20 female faces) (Fu et al., 2012). All the faces displayed neutral expressions and were unknown to the participants. Face stimuli were masked with an oval shape that hid external features (hair, ears, and jawline) using Adobe Photoshop CS5 (Adobe Systems, San Jose, CA).

**Figure 1:**
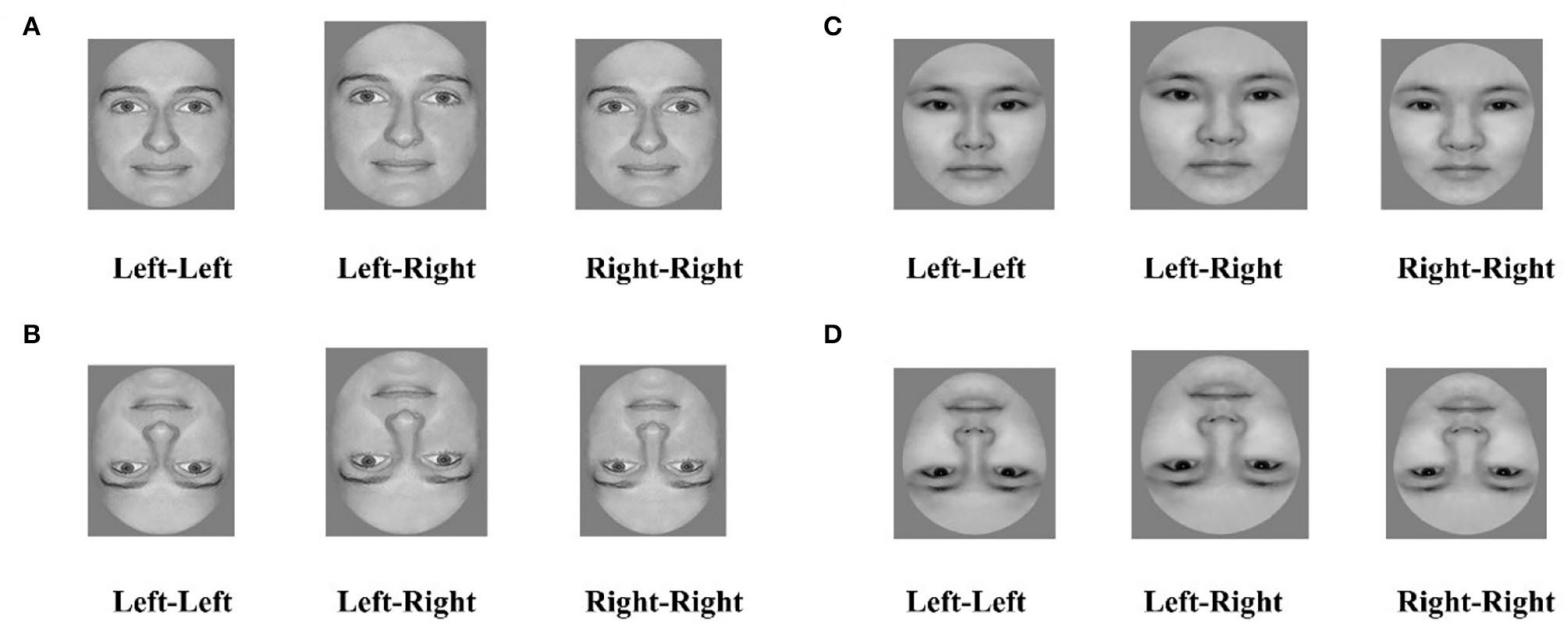
Examples of face stimuli: a “Left-Left;” Chimera made from the left-face half; the “Left-Right” original face; and a “Right-Right” chimera made from the right face half. The faces above are as follows: **(A)** upright Caucasian, **(B)** inverted Caucasian, **(C)** upright Chinese, **(D)** inverted Chinese. All individuals whose face images were used permitted us to use their photographs in academic publications.

Each original face was split vertically into left and right halves to create two chimeric faces as follows: one composed of the left half of the original face and its mirror image, and the other composed of the right half of the original face and its mirror image (Figures 1A,C). Inverted chimeras were created by flipping the upright chimeras vertically, producing 160 chimeric faces from 40 original faces (Figures 1B,D).

The original stimuli subtended visual angles of 6.3° × 7.3° when viewed from a distance of 55 cm. To discourage the participants from adopting a feature comparison or pixel-wise matching strategy (Li et al., 2021), the area of chimeric stimuli was decreased by 10%, relative to the original faces.

To evaluate brightness differences in different-race chimeric face images, we compared the different levels of brightness in left and right chimeric face images made from the same original faces, pixel by pixel, using GIMP software (GNU Image Manipulation Program). From this, we obtained mean brightness differences between the chimeras and original pictures. As the brightness difference of one Caucasian face image exceeded three standard deviations, trials involving this image were eliminated. To balance the number of Caucasian and Chinese face images, we deleted trials of the Chinese chimeric face image with the largest brightness difference. For the remaining 78 face images, independent sample *t*-tests revealed no difference between the Caucasian chimeric face images (*M* ±*SD* = 0.025 ± 0.005, range: 0.017–0.038) and the Chinese chimeric face images (*M* ±*SD* = 0.024 ± 0.007, range: 0.013–0.033), *t*_(76)_ = 1.16, *p* = 0.25, and Cohen's *d* = 0.23.

### Procedure

The participants were seated in an experimental cabin around 55 cm from a 17-inch cathode-ray tube (CRT) monitor (1,024 × 768 pixel resolution; 60 Hz refresh rate), with their heads supported by chin rests. All stimuli were viewed against a gray background, and the stimulus presentation and recording of responses were controlled by E-Prime 3.0 (Psychology Software Tools, Tools, Pittsburgh, PA).

**Figure 2:**
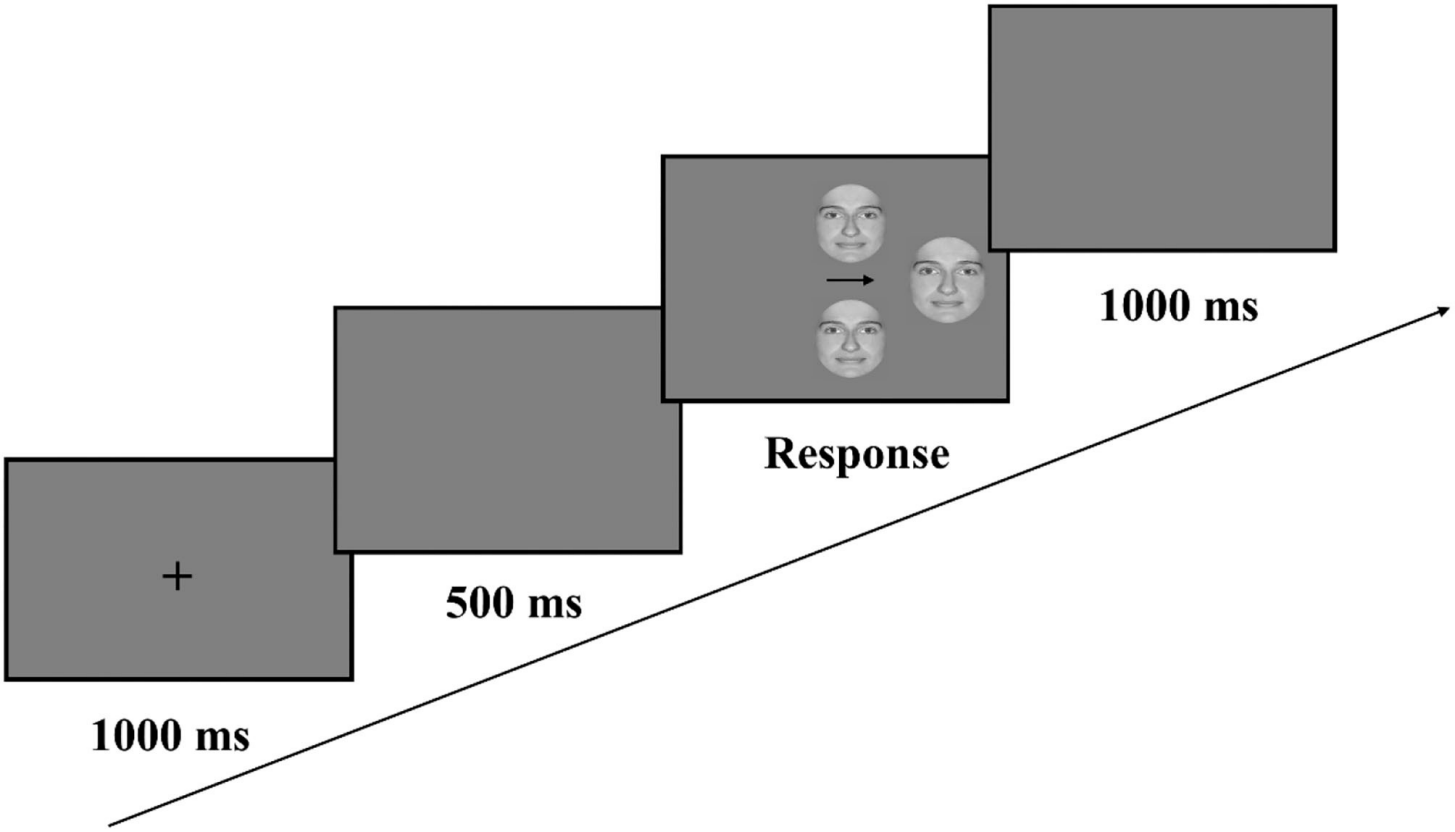
Example of an upright Caucasian chimeric face trial.

Figure 2 presents the trial scheme. Each trial began with a central fixation cross, presented for 1 s, followed by a blank gray screen, presented for 500 ms. The original face and its left and right chimeras were then presented simultaneously. The chimeras were randomly assigned to placements above or below a central arrow pointing toward the original face; they were then randomly presented on the left or right side of the screen. The centers of the chimeric images were ~6.48° from the center of the screen, while the center of the original face was ~10.65° from the center of the screen. The three faces remained onscreen until the participants responded. After the response, the screen went blank for an intertrial interval of 1 s, after which the next trial began.

The participants had to decide which of the two chimeric faces resembled the original face more closely. They were instructed to view the original face first, followed by the two chimeric faces. They then made their judgments quickly, based on first impressions. Responses were made using a standard computer keyboard. Half of the participants pressed the “T” or “N” keys with their left or right index fingers, indicating that the top or bottom chimera, respectively, resembled the original face more closely. The other half pressed the “U” or “V” keys with their right or left index fingers to select the top or bottom chimeras, respectively. This arrangement ensured that the positions of the chimera image and response key were spatially compatible, while the assigned responding hand and response key/chimera position were counterbalanced. All participants were randomly assigned to one of the two groups and were presented with upright or inverted chimeras. Both groups were presented with chimeric Chinese and Caucasian faces. Each participant worked on 320 trials, divided into eight blocks, of which four were Chinese and four were Caucasian. The order of the Chinese and Caucasian face blocks was balanced across the participants. Each block included four types of trials: the left and right locations of the original faces and the top and bottom locations of the left and right chimeras (10 trials per type). Each original stimulus was presented once per block. Before the experiment, the participants completed 16 practice trials. The entire experiment lasted ~1 h.

### Design and Data Analysis

A two-factor mixed design was used for the analysis of variance (ANOVA), with a between-subject factor orientation (upright vs. inverted) and within-subject factor race (Chinese vs. Caucasian). The dependent variable was the selection ratio of similarity between the original and left chimeric faces, calculated as the number of trials in which the participant chose the left chimeric face divided by the total number of trials; hence, selection ratios > 0.5 reflected a left-side bias (Butler and Harvey, 2005; Li and Cao, 2017). Trials with reaction times < 500 ms or >3 SD above each participant's mean reaction time were excluded. The mean proportion of the excluded trials was 1.74%. Three participants were excluded because their mean reaction time was >3 SD from the group mean. Ultimately, data from 62 participants were analyzed, with 32 participants using images in the upright condition and 30 in the inverted condition.

To examine the presence of left-side bias in the four experimental conditions, a one-sample *t*-test was conducted to compare the selection ratio in each experimental condition with the no-bias level (0.5).

We also calculated the reaction times between stimulus presentation and response separately for the left and right chimeric faces (refer to Supplementary Material).

## Results

**Figure 3:**
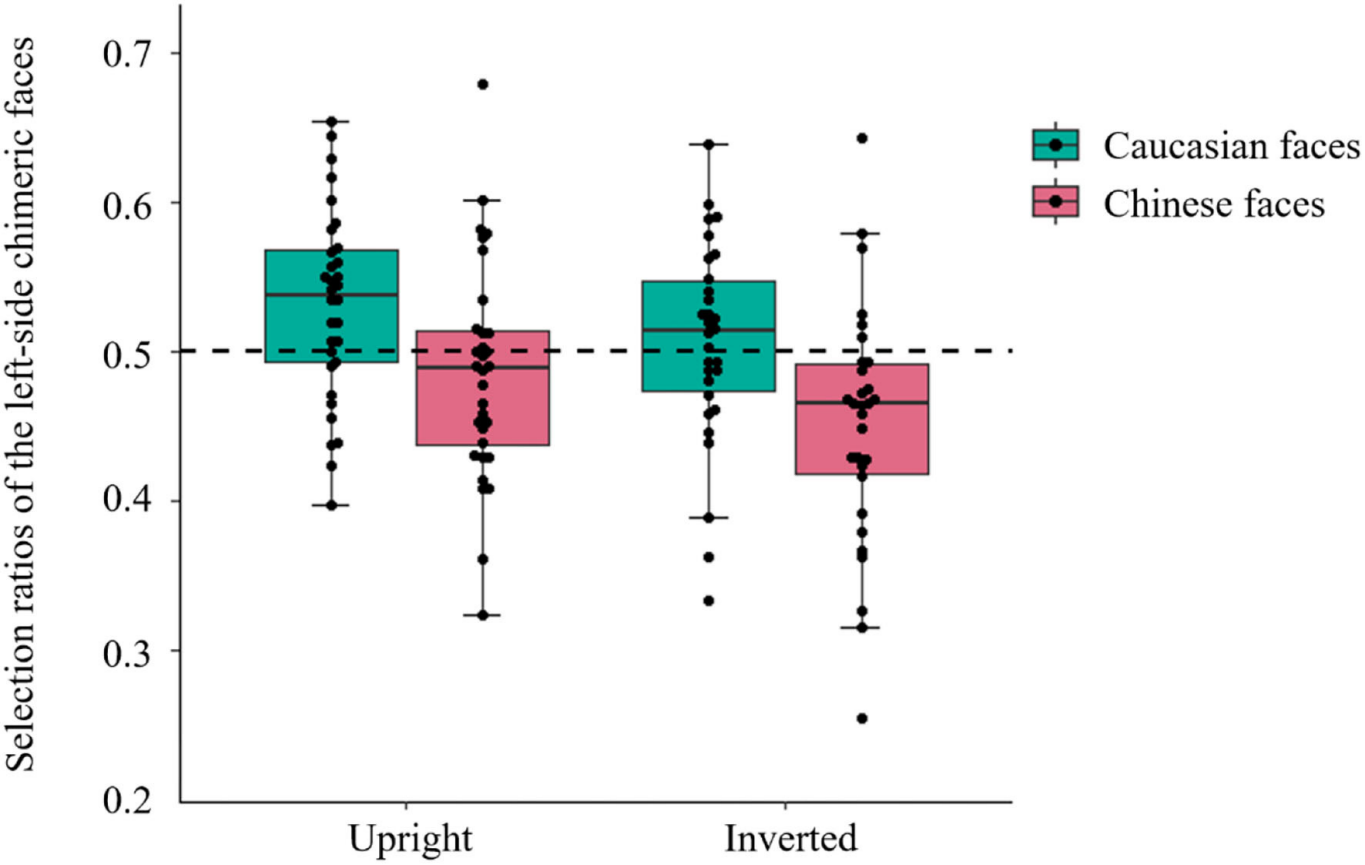
Box plots of the selection ratios for left-side vs. right-side chimeric faces in each condition. The dashed line indicates an equal selection ratio for left-side and right-side chimeric faces. Each dot represents an individual participant.

The selection ratios for the left-side chimeras are shown in Figure 3. We conducted a 2 × 2 ANOVA of the selection ratio for the left-side chimeric faces. The main effect of race was significant, *F*_(1,60)_ = 21.67, *p* < 0.001, $\eta_{p}^{2}$ = 0.27, with higher selection ratios for Caucasian (*M* ± *SD* = 0.52 ± 0.07) than Chinese faces (*M* ± *SD* = 0.47 ± 0.08). The main effect of orientation was also significant, *F*_(1,60)_ = 4.35, *p* = 0.041, $\eta_{p}^{2}$ = 0.07, with higher selection ratios in the upright condition (*M* ±*SD* = 0.51 ± 0.07) than in the inverted condition (*M* ± *SD* = 0.48 ± 0.08). The interaction between race and orientation was not significant [*F*_(1,60)_ = 0.40, *p* = 0.529, $\eta_{p}^{2}$ = 0.007].

To examine the presence of a left-side bias in each experimental condition, we conducted one-sample *t*-test to compare the selection ratio with the no-bias level (0.5). In the upright condition, there was a reliable left-side bias for Caucasian faces (*M* ±*SD* = 0.53 ± 0.06), *t*_(31)_ = 2.86, *p* = 0.008, and Cohen's *d* = 0.51 but not for Chinese faces (*M* ± *SD* = 0.49 ± 0.07), *t*_(31)_ = 0.81, *p* = 0.484, and Cohen's *d* = 0.15. In the inverted condition, there was no bias for Caucasian faces (*M* ±*SD* = 0.51 ± 0.07), *t*_(29)_ = 0.71, *p* = 0.484, and Cohen's *d* = 0.13 but a significant right-side bias for Chinese faces (*M* ±*SD* = 0.45 ± 0.08), *t*_(29)_ = 3.31, *p* = 0.002, and Cohen's *d* = 0.61.

## Discussion

The left-side bias effect is a robust behavioral marker of perceptual expertise in face perception (Hsiao and Cottrell, 2009). This present study has investigated the extent to which Caucasian individuals display left-side bias when processing upright and inverted own-race and other-race faces. As expected, we found a larger left-side bias for upright own-race faces than for other-race faces. Similar results have been reported in previous studies, which have used face stimuli with different levels of familiarity, for example, by comparing familiar and unfamiliar adult faces (Brady et al., 2005) or own-age and other-age faces (Proietti et al., 2015). Therefore, these results extend the left-side bias to another face-experience-related dimension, namely, own-race faces vs. other-race faces, providing further evidence to support the view that the left-side bias effect is influenced by the experience of face stimuli.

Importantly, this study has demonstrated that the left-side bias effect disappears when Caucasian participants process inverted own-race faces. This finding is consistent with previous studies, which have shown that face inversion eliminates the left-side bias of Caucasian observers processing Caucasian faces (Bourne, 2008, 2011; Coolican et al., 2008; Harrison and Strother, 2019; but reduced in Butler and Harvey, 2005). Furthermore, using the same paradigm as in this study, Li et al. (2021) have found that Chinese participants display no left-side bias when processing inverted own-race faces. Together, these results suggest that the absence of left-side bias during the processing of inverted own-race faces can be generalized across Chinese and Caucasian participants. During inverted face processing, the neural correlates differ from those in upright face processing. In particular, the configural/holistic processing experienced during upright face recognition is strongly impeded when inverted faces are used (McCarthy, 1999; Maurer et al., 2002). As face inversion does not change the symmetry of the image along the vertical axis or any of the local image properties of individual facial features (i.e., local contrasts between eyes and mouth), the loss of left-side bias after inversion cannot be explained by such factors. Instead, left-side bias in upright faces may reflect right hemisphere domination of holistic/configural face processing (Butler and Harvey, 2005; Bourne, 2011). Therefore, it is plausible to assume that, if information extracted from the original face is dominated by its left side (within the viewer's left visual field/right hemisphere), the left-face chimera has the advantage of being perceived as resembling the original face more closely than the right-face chimera. Now, if the left visual field/right hemisphere processing is more holistic/configural than the right visual field/left hemisphere processing, it is also plausible that impeding configural processing *via* inversion may diminish or eliminate left-side bias. This is the pattern observed in relation to inverted own-race faces, which showed no left-side bias in this study for Caucasian participants/faces or Chinese participants/faces (Li et al., 2021).

This study has identified a left-side bias among Caucasian participants for upright own-race faces only, not for upright other-race faces. These results are consistent with those of Rhodes et al. (1990), who found a left-side bias among Caucasian participants for upright own-race faces but not for upright other-race faces. Among Chinese participants, however, a left-side bias has been observed for both own- and other-race upright faces (Rhodes et al., 1990; Li et al., 2021). Together with the evidence noted above, this suggests that left-side bias may be a universal effect only in relation to upright own-race face processing; it may not always apply to upright other-race faces. However, the absence of a left-side bias in upright other-race faces must be interpreted with caution. For example, a previous study, which used inverted faces as a control condition, found that inversion primarily affected performance on the left side of a face and not on the right (Harrison and Strother, 2019).

Why was there no left-side bias for upright other-race faces among the Caucasian participants? The Chinese and Caucasian participants in the studies noted above may have had different levels of other-race face experiences. For example, the Eastern participants in China (Li et al., 2021) and Singapore (Rhodes et al., 1990) may have had more experience with other-race faces than the Caucasian participants in New Zealand (Rhodes et al., 1990) or Germany in this study. This hypothesis could be tested by asking Caucasian participants with different levels of experience to view other-race faces. Alternatively, holistic/configural face-processing skills may be stronger among Asians than Caucasians. It has been suggested that Japanese people have better configural face-processing skills than Caucasian Americans (Miyamoto et al., 2011). Importantly, the Asian participants performed better than the Caucasian participants when processing other-race faces in the face-inversion (Rhodes et al., 1989) and composite-face tasks (Michel et al., 2006). The Caucasian participants' reduced ability to process other-race faces may attenuate or eliminate their left-side bias when processing Chinese faces. The third explanation may be linked to the script systems of Eastern and Western participants. For example, Megreya and Havard (2011) compared native readers of right-to-left Arabic script with native readers of left-to-right English, showing that reading direction influenced left-side bias in face perception. However, both the German script learned by participants in this study and the Chinese script learned by previous study participants (Li et al., 2021) are read left to right. In contrast, learning to read Chinese script appears to have a specific impact on the holistic processing of faces, regardless of race, when compared to learning to read German script (Ma et al., 2022). Future studies may thus investigate how reading affects the left-side bias for same and other-race faces among Eastern and Western individuals.

In this study, the finding that a stronger left-side bias exists for Caucasian faces than for Chinese ones may indicate a Caucasian face advantage for left-side bias, not an own-race face advantage. Interestingly, Rhodes et al. (1990) found that both Caucasian and Chinese participants experienced a stronger left-side bias effect for Caucasian rather than Chinese faces, while Li et al. (2021) found that Chinese participants showed a stronger left-side bias for Caucasian rather than for Chinese faces. Together, the results of Rhodes et al.'s (1990) and Li et al.'s (2021) studies, and those of this study, indicate a Caucasian face advantage in relation to left-side bias. The Caucasian face advantage may suggest that Caucasian faces have special characteristics that induce a stronger left-side bias, in terms of symmetry or brightness, for example. As this study controlled for differences in brightness between the chimeras, we checked facial asymmetry as a potentially relevant factor, using a structural similarity algorithm to compare the symmetry of the original Chinese and Caucasian faces in the study. The algorithm divided the task of measuring similarity into three comparisons (luminance, contrast, and structure) and compared favorably with other methods in accounting for experimental measurements of subjective quality (Wang et al., 2004). The results showed that the Chinese faces in this study (*M*_Chinesefaces_ = 0.91 ± 0.007) were more symmetrical than the Caucasian faces [*M*_Caucasianfaces_ = 0.88 ± 0.007, *t*_(39)_ = 18.48, *p* < 0.001, Cohen's *d* = 2.96]. Thus, the stronger, stable left-side bias effect for Caucasian faces may reflect their more pronounced asymmetry. In other words, when human faces are viewed as largely symmetrical stimuli (Wolff, 1933; Güntürkün, 1991), symmetry may serve as an important cue in face perception (e.g., Rhodes et al., 2001, 2005; Chen et al., 2007), impacting left-side bias. Accordingly, when the participants processed Caucasian faces with less symmetrical features, they may have relied more on left-side information, inducing a left-side bias. Future studies should examine this hypothesis to determine whether the degree of facial asymmetry modulates the left-side bias effect.

In addition, our results revealed an unexpected bias toward the right side of inverted other-race (Chinese) faces. A similar pattern was found in a study involving children aged 5 years, in which Balas and Moulson (2011) explained their results as bilaterality developed early on and right lateralization developed with age. Although this study tested healthy adults with mature face perception, Li et al. (2021) found a right-side bias for own-race faces when the original face was presented in the right visual field/left hemisphere. Hence, the right-side bias for inverted faces was not specific to other-race facial features. This may reflect the fact that inverted faces cannot be processed holistically (Crookes et al., 2013; Hills et al., 2016) but require feature-based processing. To process inverted faces, when holistic strategies cannot be applied, participants may resort to analytic strategies by focusing on certain features in the original face (e.g., eyebrow slant and lip curvature) and matching them with the chimeras. Such analyses may work better in the right visual field, as the left hemisphere may be specialized in analytic processing. In this study, the deployment of analytic vs. holistic strategies was facilitated by the exclusive presentation of inverted faces in just one participant group. Li et al. (2021) and this study used the same stimuli; in both cases, a right-side bias was observed for the Chinese stimuli. Thus, face inversion may have facilitated an analytical, feature-based processing strategy, impeding the deployment of holistic processing and reflecting a high degree of facial symmetry in the Chinese faces.

Interestingly, the vast majority of participants in previous and present studies appear to have been right-handed (Gilbert and Bakan, 1973; Campbell, 1978; Heller and Levy, 1981; Levy et al., 1983). Left-handed individuals have been shown to have smaller perceptual biases for face chimeras than right-handers (Gilbert and Bakan, 1973; Levy et al., 1983; Hoptman and Levy, 1988; Luh et al., 1994). Consequently, handedness may have a potential influence on left-side face bias effects. Future studies should examine whether there are any differences in facial left-side bias between right- and left-handed individuals.

## Conclusion

This study has demonstrated a stronger left-side bias for own-race faces than for other-race faces in Caucasian adults, suggesting that the extent of left-side bias diminishes when research participants have less experience of (other-race) faces. This study clearly reveals that Caucasian participants experience no left-side bias when own-race faces are processed in an inverted orientation, suggesting that the absence of left-side bias during the processing of inverted own-race faces is a race-independent phenomenon. Interestingly, the results reveal no left-side bias when Caucasian participants process upright other-race faces, suggesting that left-side bias may be a universal effect for upright own-race faces but not for upright other-race faces.

## Data Availability Statement

The raw data supporting the conclusions of this article will be made available by the authors, without undue reservation.

## Ethics Statement

The studies involving human participants were reviewed and approved by Zhejiang Normal University Ethics Committee. The patients/participants provided their written informed consent to participate in this study.

## Author Contributions

JK and XC: designed the experiments. JK: executed the project. JK, CL, and XC: performed the data analysis. JK, CL, WS, and XC: wrote the manuscript and revised the manuscript. All authors reviewed the manuscript. All authors contributed to the article and approved the submitted version.

## Funding

This study was supported by the National Social Science Foundation of China (Grant No. 21FYYB051) and the Sailing plan for graduate students at Zhejiang Normal University.

## Conflict of Interest

The authors declare that the research was conducted in the absence of any commercial or financial relationships that could be construed as a potential conflict of interest.

## Publisher's Note

All claims expressed in this article are solely those of the authors and do not necessarily represent those of their affiliated organizations, or those of the publisher, the editors and the reviewers. Any product that may be evaluated in this article, or claim that may be made by its manufacturer, is not guaranteed or endorsed by the publisher.
