# Supplementary material for: The Left-Side Bias Is Reduced to Other-Race Faces in Caucasian Individuals
Source: Front Psychol. 2022 Apr 25;13:855413. doi: 10.3389/fpsyg.2022.855413 (PMC9083412; doi:10.3389/fpsyg.2022.855413)
Supplement: Supplementary file 1 [file Table_1.DOCX]

**Supplementary material**

**Analysis of Reaction times**

The mean reaction times for the selection of left and right chimeric faces in each condition are given in Table 1. ANOVA with between-subject factor orientation (upright, inverted) and within-subject factors face race (Chinese face, Caucasian face) and selection type of chimeric face (left chimeric face, right chimeric face) was conducted on mean reaction times. No main effects or two-way interactions were found. A significant three-way interaction among all factors was found, *F* (1, 60) = 6.16, *p* = .016, *η_p_^2^* = .10. Further analyses were performed separately for each orientation group with factors face race and selection type. In upright condition, the interaction between the selection type and face race was significant, *F* (1, 31) = 10.30, *p* = .003, *η_p_^2^* = .25. Post-hoc comparisons revealed that there was no difference between the selection of left- and right-chimeric Caucasian faces (*M* *± SD* = 2235 ± 875 vs. 2280 ± 979 ms), *t* (31) = 1.37, *p* = .181, Cohen’s *d* = .04, but shorter reaction times for the selection of right than left chimeric Chinese faces (*M* *± SD* = 2230 ± 849 vs. 2146 ± 749 ms), *t* (31) = 2.38, *p* = .024, Cohen’s *d* = .09. In inverted condition, there was only a significant effect of the selection type of chimeric faces, *F* (1, 29) = 4.38, *p* = .045, *η_p_^2^* = .13, with shorter reaction times for right than left chimeric faces (*M* *± SD* = 2371 ± 836 vs. 2324 ± 2324 ms), *t* (29) = 2.09, *p* = .045, Cohen’s *d* = .05.

Table 1: Mean reaction times (ms) for the selection of left and right chimeric faces

|  | Upright | | Inverted | |
| --- | --- | --- | --- | --- |
| The selection type of chimeric face | Caucasian face | Chinese face | Caucasian face | Chinese face |
| Left chimeric faces | 2235 ± 875 | 2230 ± 849 | 2382 ± 953 | 2360 ± 847 |
| Right chimeric faces | 2280 ± 979 | 2146 ± 749 | 2326 ± 902 | 2322 ± 800 |
